# Virulence Factors as Targets for Anticryptococcal Therapy

**DOI:** 10.3390/jof2040029

**Published:** 2016-11-30

**Authors:** Renata V. D. M. Azevedo, Juliana Rizzo, Marcio L. Rodrigues

**Affiliations:** 1 Fundação Oswaldo Cruz—Fiocruz, Centro de Desenvolvimento Tecnológico em Saúde (CDTS), 21040-361 Rio de Janeiro, Brazil; marciolr@cdts.fiocruz.br; 2 Instituto de Microbiologia Paulo de Góes (IMPG), Universidade Federal do Rio de Janeiro, 21941-902 Rio de Janeiro, Brazil; juju.rizzo@gmail.com; 3 Instituto de Bioquímica Médica (IBqM), Universidade Federal do Rio de Janeiro, 21941-902 Rio de Janeiro, Brazil

**Keywords:** *C. neoformans*, cryptococcosis, virulence factors, anticryptococcal targets

## Abstract

The global mortality due to cryptococcosis caused by *Cryptococcus neoformans* or *C. gattii* is unacceptably high. Currently available therapies are decades old and may be impacted by drug resistance. Therefore, the need for more effective antifungal drugs for cryptococcosis is evident. A number of *Cryptococcus* virulence factors have been studied in detail, providing crucial information about the fungal biology and putative molecular targets for antifungals. This review focuses on the use of well-described virulence factors of *Cryptococcus* as potential anticryptococcal agents.

## 1. Introduction

### 1.1. The Need for Antifungal Drugs

The number of invasive fungal infections has increased during the last few decades. About 1.2 billion people worldwide are estimated to suffer from fungal diseases [1,2] and the mortality rates for invasive infections are inadmissibly high [1]. In Brazil, a recent study estimated that serious fungal diseases affect more than 3.8 million people [3]. It is now clear that the number of deaths caused by fungal infections is similar to those produced by either tuberculosis [4] or malaria [5]. 

The great majority of reported fungal related deaths result from infections by four genera of fungi: *Aspergillus*, *Candida*, *Cryptococcus*, and *Pneumocystis* [1,6]. The limiting factors in the treatment of fungal infections comprise late diagnosis, high cost, and also drug-related issues, which include limited routes of administration, high toxicity, reduced spectrum of activity, unfavorable interactions, resistance, and reduced bioavailability in target tissues [7,8]. 

Despite the increasing necessity for new antifungals, currently available drugs are limited in number [9] and not many are in preclinical development [7,10,11]. The long timeline required for drug development has stimulated drug-repositioning approaches. Sertraline, the most frequently prescribed antidepressant, is in clinical trial for cryptococcosis [12,13]. The drug has demonstrated in vivo antifungal activity in experimental cryptococcosis when administered either alone or synergistically with fluconazole [12]. Moreover, sertraline treatment prevented paradoxical cryptococcal immune reconstitution inflammatory syndrome (CM-IRIS) and relapse [14].

### 1.2. Cryptococcosis: A Systemic Fungal Infection with Alarming Mortality Indices

*C. neoformans* and *C. gattii* are encapsulated yeast-like pathogens causing human and animal cryptococcosis. Humans are exposed to *Cryptococcus* by inhalation of environmental infectious propagules [15,16]. Cryptococcosis has a worldwide distribution and is predominantly associated with immunocompromised individuals [17], although immunocompetent hosts can also acquire the disease [18]. Usually, the infection is asymptomatic and restricted to the lungs in individuals without impaired immunity. However, in immunocompromised patients the yeast cells can widely disseminate to most organs and commonly cause pneumonia and meningoencephalitis [19]. Human cryptococcosis became a global health problem in the 1980s in concomitance with the AIDS epidemics [17,19]. Cryptococcal disease is the second leading cause of mortality in HIV patients [19].

### 1.3. Potential Targets for Development of Novel Anticryptococcal Agents

The emergence of multi-resistant microbes points to the need of new strategies to develop novel antifungal therapies. An alternative approach to conventional antimicrobial therapy is to target virulence factors required to cause host damage and disease. As reviewed by Clatworthy and colleagues [20], this approach has several potential advantages, including expanding the repertoire of microbial targets, preserving the host endogenous microbiome, and exerting less selective pressure, which may result in decreased resistance. A number of *C. neoformans* virulence factors have been studied in detail and molecularly characterized. The main virulence-related molecules include capsular polysaccharides, melanin, and secreted enzymes [21,22,23,24,25]. Below, we describe *C. neoformans* virulence factors showing potential as targets for the development of novel antifungal drugs. 

#### 1.3.1. Capsule Components

One of the most important features of *Cryptococcus* is its outermost polysaccharide capsule, which protects the fungal cell from an array of host defense mechanisms [26,27]. The importance of the capsule for virulence is clear, since acapsular mutants are clearly less virulent than wild type strains [28,29]. The molecular composition of the polysaccharide capsule includes glucuronoxylomannan (GXM) and glucuronoxylomannogalactan (GXMGal), which assume a complex spatial conformation [30]. 

Despite the poor immunogenicity of capsular polysaccharides, monoclonal antibodies (mAb) against capsule components were successfully generated through the use of conjugate antigens [31,32]. Some of the capsule mAbs were shown to bind all *C. neoformans* serotypes, with consequent activation of the complement pathway and enhancement of the antifungal activity of immune cells [32]. Passive administration of GXM-binding mAbs prolonged survival of lethally infected mice [33]. However, administration of IgG3 enhanced the infection and shortened the lives of immunocompetent mice [34] and also of CD4- and C5-deficient animals [33,34]. Phase I tests with the GXM-binding mAb 18B7 revealed undesired effects at doses higher than 1 mg/kg [35].

Alternative strategies to control cryptococcal virulence could involve the enzymes required for capsule biosynthesis. Several glycosyltransferases are necessary for synthesis and assembly of GXM and GXMGal backbones [36]. 

The first capsule-related enzyme identified was cryptococcal xylosyltransferase 1 (Cxt1), a large transmembrane protein which mediates β-1,2-linkage formation by transferring xylose from a donor nucleotide to a reducing mannose acceptor [37,38]. Deletion of the corresponding gene affected fungal survival in the lungs of infected mice, indicating that the presence of xylosyle linkages in the capsule is important for host-pathogen interactions [39]. Genome analysis revealed the existence of five homologs of *CXT1* in *C. neoformans* and 34 orthologs in other fungi, but none in other organisms [37]. Xylosyl transfer in *C. neoformans* also requires xylosyltransferase 2 (Cxt2), which also mediates β-1,2-xylose addition to mannosyl units [40]. However, contrary to the closely related Cxt1, little is known regarding the functional role of Cxt2. Although xylosyltransferase activity is present in many organisms, including plants and animals [41,42], Cxt1 and its homologous counterparts comprise a novel family of glycosyltransferases which is unique to the fungal kingdom and, therefore, represent attractive targets for selective and effective therapeutic agents [37].

Reilly and colleagues have recently described that the addition of xylose-phosphate (Xyl-P) to mannose-containing substrates, generating xylosylphosphomannose (Xyl-P-Man), also occurs in *C. neoformans* [40]. The enzyme responsible for this activity was xylosylphosphotransferase 1 (Xpt1), which does not resemble any other known xylosyltransferase. So far, this enzymatic activity has only been observed in *C. neoformans* and its biological significance is still under investigation. A mutant strain (*xpt1Δ*) revealed that the loss of Xpt1 did not influence the ability of *C. neoformans* to colonize mice lungs, express virulence factors, and grow under stress conditions [43], although one cannot discard its role in virulence before other infection models can be assessed. Interestingly, no capsular polysaccharides seem to contain the Xyl-P-Man moiety, suggesting that this enzyme may be involved in other metabolic processes, such as *O*-glycosylation of proteins [43]. The exclusive occurrence in *C. neoformans* suggests that future studies aiming at elucidating the functions of Xpt1 may produce important information on unusual glycans and their biosynthetic pathways.

Enzymes involved in mannose incorporation to capsular polysaccharides were also studied in detail [44,45]. Mannosyl units represent more than 50% of the capsule mass [46]. This carbohydrate unit is coupled with GDP (Guanosine diphosphate) for utilization in glycan biosynthetic reactions [46]. Most GDP-mannose donors are used within the lumen of Golgi complex, thus requiring specific transmembrane transporters to leave the cytosol and reach this organelle. In *C. neoformans*, there are two GDP-mannose transporters, Gmt1 and Gmt2, which are similar at the amino acid level but distinct in cellular function [44]. While both factors are able to promote GDP-mannose transport in vitro and to effectively complement its homolog in *S. cerevisiae*, the phenotypic outcome of *C. neoformans* single mutations revealed significant differences in cellular growth, colony morphology, protein glycosylation, and capsule structure [44,45]. In addition, transcription analysis revealed that they are not coordinately regulated, providing supporting evidence that their biological roles are distinct, at least to some extent [44]. Further investigation with a double mutant strain lacking *Gmt1* and *Gmt2* revealed that capsule biosynthesis, protein glycosylation processes, and virulence were severely affected [45]. GDP-mannose transporters have also been identified in other fungi [47,48,49,50,51], plants [52,53], and the protozoan parasite *Leishmania donovani* [54]. However, humans and other mammalian hosts of *C. neoformans* do not perform mannosylation reactions inside the Golgi lumen and therefore do not express any form of GDP-mannose transporters, indicating that these proteins may be potential targets for antifungal chemotherapy [45].

Mannosyltransferases constitute another class of enzymes involved in mannose incorporation into the capsule [55]. In *C. neoformans*, Cmt1 is the enzyme generating the α-1,3-linked backbone of the GXM polymer [56]. Deletion of *CMT1* abolished the enzymatic activity but did not prevent capsule formation or virulence in an infected mouse model, suggesting that other factors might also participate in GXM biosynthesis, compensating for the loss of Cmt1 [56]. A possible candidate is the protein encoded by *CAP64*, which was found to interact with Cmt1 [56]. Interestingly, the search for similar proteins in non-redundant databases, including the model yeast *S. cerevisiae*, yielded a Cmt1 homolog, the predicted product of *C. neoformans CAP59* gene [56]. Additional homologs in other species may also exist, as suggested in the dimorphic fungus *Paracoccidioides brasiliensis* [57]. Finally, mammals and other animal hosts lack Cmt1, which seems to be found exclusively in fungal species. Thus, despite the non-conclusive data, Cmt1 and associated enzymes should not be ruled out as putative targets for therapeutic intervention. 

Recently, through a proteomics-based approach, a hydrolytic enzyme involved in capsule assembly was identified as lactonohydrolase (Lhc1) [58]. This secreted enzyme is believed to affect the tertiary structure of the capsule, since mutants lacking the gene encoding Lhc1 exhibited larger capsules with altered branching, density, and solvation [58]. Most importantly, the *lhc1Δ* strain showed increased antibody opsonization and decreased survival when incubated with macrophage-like cells, as well as reduced virulence in mice [58]. Finally, lacnotohydrolases are commonly found in plants, fungi, and bacteria, but never have been described in humans (the closer relatives are the serum paraoxonases) [59], which makes this enzyme an interesting target for antifungal compounds.

#### 1.3.2. Melanin

Melanin contributes to *C. neoformans* virulence by increasing its survival in macrophages and by protecting the fungus against host effector mechanisms, such as production of oxidants and microbicidal peptides [60]. Melanized fungal cells have lower susceptibility to amphotericin B and caspofugin, interfering with the effectiveness of the treatment for cryptococcosis [61]. 

Melanin is an attractive target for therapeutic intervention. Compounds binding the pigment with high-affinity have been identified and tested for their ability to affect cellular growth, viability, pigmentation, and capacity to prolong survival of infected mice [62,63]. One of these compounds, the anti-psychotic drug trifluoperazine, effectively killed melanized cells in vitro and in vivo [63,64]. Another example is the herbicide glyphosate, which inhibited melanin production and prolonged survival of lethally infected mice [65]. The anti-parasitic drug chloroquine, on the other hand, was able to bind melanin and promote toxic activity on human melanoma cells [66], but produced no inhibitory outcome on fungal cells [63]. Despite these promising data, melanin-binding drugs have not been tested in humans.

Melanin can also be targeted by binding peptides or specific antibodies [67]. When tested in lethally infected mice, mAbs to melanin prolonged survival of infected mice and reduced fungal burden in different organs [67], suggesting that passive immunization with melanin mAbs could have therapeutic potential. Additionally, since this pigment is present in many fungal species, mAbs could be used to treat diseases other than cryptococcosis. 

In *C. neoformans*, the pigment melanin is synthesized by the enzyme laccase, a glycosylated copper-containing protein with the ability to oxidize diphenolic substrates acquired extracellularly [68]. In addition to melanin, laccase is able to generate other products, such as dopamine *O*-quinone, which is spontaneously converted to dopaminochrome, a cytotoxic compound in the brain [69]. Moreover, this enzyme possesses iron oxidase activity, which was shown to reduce hydroxyl radical formation in macrophages [70]. *LAC1* and *LAC2* are *C. neoformans* genes encoding laccases, which are positioned 8 kb apart from each other [71]. The corresponding proteins differ in subcellular location, with Lac1 being predominantly found on the cell wall and Lac2 in the cytoplasm [71,72]. Deletion mutants of both genes showed reduced melanin production, although *LAC1* seems to play a major role in pigmentation [71]. The absence of *LAC1* also resulted in diminished virulence and prolonged survival in mice [73], which was not replicated in *LAC2* deficient strains [74]. *LAC1*’s predominant role in infection was supported by the observation that *lac1Δ* mutants had decreased ability to escape the oxidative attack of primary macrophages. In this model, *lac1Δ* mutants were affected more severely (i.e., higher killing index) than *lac2Δ* cells. An important additive role played by both factors was suggested based on the greater response achieved in *lac1Δlac2Δ* double mutants [71]. Given the importance of laccase for melanin production and other virulence mechanisms, combined with the fact that similar enzymes are absent in the human host, this enzyme is an obvious target for antifungal therapies.

#### 1.3.3. Extracellular Enzymes

Secretion of extracellular enzymes is crucial for *C. neoformans* virulence [75]. Extracellular hydrolases are necessary for obtaining essential nutrients from the surrounding environment of *C. neoformans*. Therefore, it is expected that this fungus will export a number of enzymes able to degrade proteins, lipids, sugars, and nucleic acids into smaller subunits or secondary metabolites to be internalized by the fungal cell. Some of these enzymes are required for virulence by mediating tissue invasion, colonization, or modulation of the immune response [76]. Others, including proteases and DNAses, have still obscure functions and will not be included in the discussion of this topic.

*C. neoformans* urease is required for urea metabolism in the environment, providing substrates to be used in different fungal metabolic pathways [77]. The enzyme is also a major virulence factor [77]. Urease contributes to the dissemination of fungal cells from the lung to the central nervous system, enhancing pathogen sequestration within microcapillary beds, thus aiding blood-brain barrier crossing [78]. Importantly, a urease knockout strain (*ure1*) was hypovirulent, yielding lower cryptococcal burden in the brain and reduced mortality rates of lethally infected mice [78]. Considering that urease is naturally absent in humans, pharmacological inhibition or antibody therapies have been proposed as useful strategies to protect the brain against cryptococcal meningitis [77,79].

*C. neoformans* also produces extracellular phosphatases, which influence adhesion to epithelial cells and promote invasion of host tissues [80]. Apparently most of the *C. neoformans* phosphatase activity comes from the acidic phosphatase Aph1 [81]. The enzyme is transported to the fungal cell periphery and vacuoles via endosome-like structures [81]. Survival assays with the wax worm *Galleria mellonella* and mice showed that animals infected with the *aph1* deletion mutant lived longer than those infected with the wild type strain (WT), demonstrating that Aph1 contributes to cryptococcal virulence [81]. It is believed that this enzyme acts by recycling phosphate from other molecules inside the vacuoles, as well as by acquiring phosphates from the extracellular environment [81]. Sequence analysis indicates that Aph1 belongs to the group of histidine acid phosphatases and is most similar to fungal phytases (phytic acid-degrading enzymes) [82]. Interestingly, while the catalytic domain seems to be conserved, the proton donor and substrate-binding residues vary considerably among these enzymes, opening room for site-specific drug inhibition strategies [82]. 

Extracellular phospholipases B and C of *C. neoformans* are also important mediators of pathogenesis. Phospholipase B, encoded by the *PLB1* gene, promotes the growth of cryptococcal cells within macrophages [83,84,85]. The enzyme is also required for interstitial pulmonary infection and further dissemination to lymph nodes and blood [86]. Under temperature stress, secretion of Plb1 decreases, resulting in enzyme accumulation on the cell wall [87]. At this cellular site, the enzyme appears to regulate wall integrity [87]. 

Phospholipases B have been described in several eukaryotes, including yeast and mammals [88]. However, Plb from pathogenic fungi seems to have unique properties, making them potential targets for antifungal drug development [89]. Many compounds have been tested against the fungal Plb. Some commercially available compounds displayed antifungal activity and were selective for *C. neoformans* Plb1, showing no inhibition of the mammalian phospholipase [90]. A *bis*-pyridinium compound with broad-spectrum fungicidal activity, while non-toxic to mammalian cells, was recently described [89,90].

Phospholipase C is encoded by two genes, namely *PLC1* and *PLC2* [91]. In contrast to *PLC2*, *PLC1* seems to be crucial for fungal growth and morphology, cell wall integrity, and antifungal drug resistance [91]. Importantly, the functions of *PLC1* are also connected to other virulence related phenotypes, such as secretion of phospholipase B, melanin production, and growth at 37 °C [91]. Plc1 biochemical functions include generation of inositol trisphosphate (IP3) as a substrate for Arg1 kinase, which is fundamental for fungal virulence [92]. *C. neoformans* Plc1 differs from the mammalian homolog Plc-δ in many aspects, although the fungal and mammalian enzymes share 32% of similarity. The mammalian enzyme possesses both the EF hand regulatory domain and the pleckstrin homology (PH) membrane binding domain that are absent in the fungal enzyme, indicating discrepancies in their mechanism of action [90]. Differences in the N-terminal length between the fungal and the mammalian enzymes were also observed [93,94]. Moreover, yeast Plc is predicted to be basic whereas the mammalian enzyme is an acidic protein. These structural differences associated with the role of Plc1 as a virulence factor reinforces phospholipase C as a potential antifungal drug target [93].

Cryptococcal cells secrete superoxide dismutases (Sods), enzymes that convert superoxide to hydrogen peroxide and oxygen, thus reducing the harmful load of oxidants in the extracellular milieu [95]. It has been reported that Sods may assist fungal growth inside macrophages by buffering superoxide molecules, which are released as part of the immune response [96]. At least two Sods are produced by *C. neoformans*, namely Sod1 and Sod2 [97,98,99]. Single and double knockout mutants showed that both are important for virulence in a mouse model of cryptococcosis. While Sod1 primarily acts by mediating resistance to superoxide attack by neutrophils, Sod2 protects fungal cells from high concentration superoxide anions that would otherwise limit growth [98,99]. Interestingly, Sod production is increased at higher temperatures, such as that inside the human host [100,101]. Despite the presence of human homologs of cryptococcal Sods [97,99], these enzymes could still be considered interesting drug targets if fungal-specific inhibitors are discovered. In fact, little variation was found in Sod2 proteins among *C. neoformans* variants, indicating that drugs affecting this potential target could be useful against all cryptococcal serotypes [101].

#### 1.3.4. Regulators of Unconventional Secretory Pathways

As previously mentioned, the pathogenesis of *C. neoformans* is incontestably dependent on secretory mechanisms, since many virulence factors are surface-associated or extracellular [102]. The conventional pathway by which eukaryotic cells secrete molecules to the cell surface requires the traffic of proteins containing signal peptides from the endoplasmatic reticulum (ER) to the Golgi [103]. The remarkable lack of signal peptides in most of the well-characterized virulence factors of *C. neoformans* suggests that unconventional secretion pathways are involved directly with cryptococcal pathogenesis.

Different regulators of unconventional secretion in *C. neoformans* have been studied, including Golgi reassembly and stacking protein (Grasp), the eukaryotic sucrose non-fermenting protein 7 (Snf7), and the putative aminophospholipid translocase (Apt1 flippase) [104,105,106]. A *C. neoformans* Grasp mutant exhibited attenuated virulence in mice in comparison to wild type cells [104]. Deletion of *GRASP* also resulted in reduced polysaccharide secretion, decreased capsular dimensions, altered Golgi morphology, and efficient association to macrophages [104]. Likewise, Xu and colleagues demonstrated that *graspΔ* mutant cells have reduced capsules and impaired hyphal growth [107]. Recently, biochemical and biophysical studies of *C. neoformans* Grasp in solution revealed the presence of intrinsically disordered regions (IDRs) in its structure. These regions can be crucial to understanding the cellular processes in which Grasp participates [108]. 

Similar to Grasp, the deletion of SNF7, the gene encoding a protein involved in multivesicular body (MVB) formation, affected crucial virulence determinants in both *C. neoformans* and *C. gattii* [106]. *snf7∆* mutants manifested pigmentation defects, reduced extracellular GXM, defective capsule formation, loss of virulence in a murine model of intranasal cryptococcal infection, and reduced survival in macrophages [106,109]. Finally, mutation of *C. neoformans APT1* resulted in defective synthesis and vesicular export of GXM, diminished survival rates in the lungs of infected mice, ineffectiveness of brain colonization, and changes in the Golgi morphology [105]. Abnormal Golgi in *apt1∆* cells is probably due to defects in the control of membrane asymmetry, the primary function of the lipid flippases [105]. Deletion of *CDC50*, which encodes the β-subunit of a lipid flippase, affected fungal virulence and survival inside macrophages [110].

#### 1.3.5. Other Cryptococcal Targets

The fungal glycosphingolipid glucosylceramide (GlcCer) is involved in a range of biological events in *C. neoformans*, including cell growth, lipid raft formation, immunogenicity, alkaline tolerance, and, ultimately, regulation of fungal virulence [111]. Although GlcCer is generally present in membranes, it accumulates on the cell wall [111,112]. Antibodies to *C. neoformans* GlcCer inhibited fungal growth in vitro [112] and prolonged the survival of lethally infected mice [113]. A *C. neoformans* mutant strain lacking GlcCer synthase (Gcs1) failed to grow in neutral/alkaline pHs that mimic the extracellular environment of the host during infection [114]. On the basis of the fact that human and fungal GlcCer are structurally different [115], drug library screenings targeting GlcCer synthesis were performed to identify GlcCer inhibitors. Two hydrazycins, BHBM and its derivative D0, interfered with GlcCer synthesis, impaired vesicular transport, and controlled animal cryptococcosis [116]. In this context, GlcCer is an already validated target for new antifungal drugs. 

Heat-shock proteins (Hsps) play important roles in various biological processes including transcription, translation, protein folding, and aggregation of proteins [117]. A recent study has demonstrated that targeting *C. neoformans* Hsp90 with radicicol (RAD) affected in vitro growth of planktonic cells of *Cryptococcus* and improved the in vitro effect of antifungal drugs, especially fluconazole [118]. The enhanced antifungal activity of fluconazole was also detected through in vivo, using *Caenorhabditis elegans* as a host [118]. The effects of RAD included reduced permeability of the plasma membrane and decreased capsular dimensions [118]. Antibody therapies targeting Hsp90 have been tested in the *C. albicans* model [119], reinforcing the potential role of this protein as a target for antibodies.

It has been demonstrated that the calcineurin pathway represents a key component on cell stress responses [120] and is involved in *C. neoformans* pathogenesis, due to its crucial role in determining fungal growth based upon the host’s temperature (37 °C) [121]. Calcineurin mutants are unable to grow at elevated host temperatures and are not pathogenic in animal models of infection [122]. Therefore, the calcineurin pathway can be a potential therapeutic target for cryptococcosis. Studies utilizing the calcineurin inhibitors cyclosporine A (CsA) and tacrolimus (FK506), both immunosuppressants, revealed the potential of the calcineurin pathway to identify inhibitors of fungal growth [121,123]. Although it is challenging to use drugs inhibiting pathways highly conserved from human to yeast, such as the calcineurin pathway and Hsp90, even the presence of slightly structural differences in the drug binding site can be used to develop target specific inhibitors [124]. Previously, subtle structural differences allowed fungal specific calcineurin inhibitor analogs to be identified [125]. Additionally, in some other cases, such as the Hsp90 chaperone complex, co-factors can be used as a target for fungal specific inhibitors [124]. 

*N*-myristoylation is an essential protein modification process catalyzed by the enzyme *N*-myristoyltransferase (NMT), which relocates myristate from myristoyl-CoA to the N-terminal glycine residue of a specific set of cellular proteins. In *C. neoformans*, a functional NMT is determinant for survival and dissemination inside the host [126,127]. The myristate analog 4-oxatetradecanoic acid was an effective NMT inhibitor associated with fungicidal effects in vitro [128]. Other NMT inhibitor compounds with putative fungicidal properties have been identified and may support the design of NMT-based therapies for cryptococcosis [129,130,131]. While *N*-myristoylation is abundant in protozoa, fungi, and mammals [132], peptide-binding studies revealed that substrates for NMTs are specific for each species. This observation indicates a great potential for development of species-specific inhibitors [132,133].

## 2. Concluding Remarks

**Figure 1:**
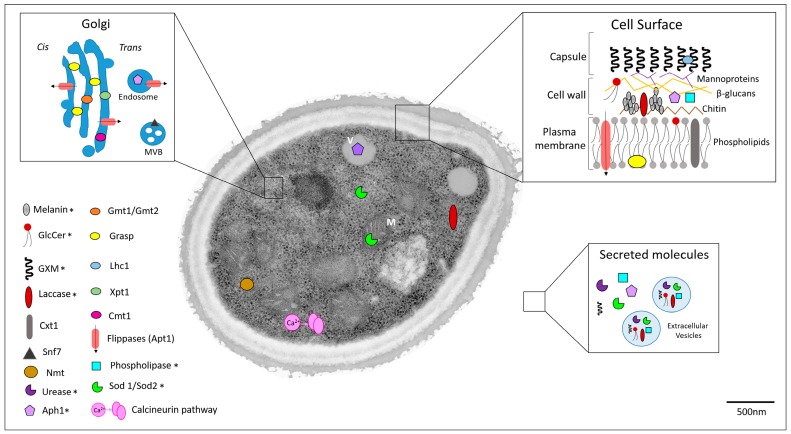
Cellular localization of potential targets for anticryptococcal therapy. Asterisks denote cellular targets that had their distribution confirmed by microscopic approaches. Other targets had their cellular distribution estimated according to their predicted or characterized functions. Cell surface, Golgi, and extracellular environment are highlighted. Secreted molecules are differentiated into soluble or extracellular-associated vesicles. **M**: mitochondria; **V**: vacuole; **MVB**: multivesicular bodies.

This review highlights several aspects related to the identification of cryptococcal virulence factors that could be used as targets for the development of new drugs, as summarized in Figure 1. The identification of new molecular targets and therapeutic strategies to fight cryptococcal disease is not only important but also necessary to improve human survival rates, especially in immunocompromised individuals. Virulence factors and associated enzymatic pathways are obvious target choices, since their functions directly affect interaction with the host and the immune response.
